# Mathematical models and deep learning for predicting the number of individuals reported to be infected with SARS-CoV-2

**DOI:** 10.1098/rsif.2020.0494

**Published:** 2020-08-05

**Authors:** A. S. Fokas, N. Dikaios, G. A. Kastis

**Affiliations:** 1 Department of Applied Mathematics and Theoretical Physics, University of Cambridge, Cambridge CB3 0WA, UK; 2 Research Center of Mathematics, Academy of Athens, Athens 11527, Greece; 3 Viterbi School of Engineering, University of Southern California, Los Angeles, CA 90089, USA; 4 Centre for Vision, Speech and Signal Processing, University of Surrey, Guildford, UK

**Keywords:** mathematical modelling of epidemics, COVID-19, Riccatti equation, Burgers' equation, integrable systems, inverse problems

## Abstract

We introduce a novel methodology for predicting the time evolution of the number of individuals in a given country reported to be infected with SARS-CoV-2. This methodology, which is based on the synergy of explicit mathematical formulae and deep learning networks, yields algorithms whose input is only the existing data in the given country of the accumulative number of individuals who are reported to be infected. The analytical formulae involve several constant parameters that were determined from the available data using an error-minimizing algorithm. The same data were also used for the training of a bidirectional long short-term memory network. We applied the above methodology to the epidemics in Italy, Spain, France, Germany, USA and Sweden. The significance of these results for evaluating the impact of easing the lockdown measures is discussed.

## 1. Introduction

The novel coronavirus SARS-CoV-2 is the third coronavirus to appear in the human population in the past two decades, following the severe acute respiratory syndrome coronavirus SARS-CoV outbreak in 2002 and the Middle East syndrome coronavirus MERS-CoV outbreak in 2012. SARS-CoV-2 initially emerged in Wuhan, China, at the end of 2019; after Chinese scientists identified the sequence of the causative virus [1], this information was immediately shared with the international community. Furthermore, China took effective measures for the containment of the spread of this outbreak. This new coronavirus is less pathogenic than the earlier two coronaviruses [2]. For example, in the first case of pneumonia caused by this virus reported in USA, a 35-year-old, healthy, individual (who had travelled in Wuhan) presented in a hospital four days after he experienced dry cough and low grade fever; he proceeded to develop pneumonia 5 days later, but quickly recovered [3]. This is the typical disease course for young persons. However, COVID-19 has a significant mortality rate for elderly persons and for those with a variety of underlying medical conditions, including respiratory and cardiovascular diseases, as well as diabetes mellitus. For example, after the identification of an individual in a skilled nursing facility in USA infected with SARS-CoV-2, extensive testing was carried out and 23 days later it was established that 57 of 89 residents (67%) were infected; as of 3 April, 11 of the infected persons had been hospitalized (three in the intensive care unit) and 15 had died (mortality 27%) [4]. A crucial factor for the high transmissibility of this virus is the high level of SARS-CoV-2 shedding in the upper respiratory tract even among asymptomatic individuals [5]. As a result of the above facts, and the lack of appropriate *early* international measures for the suppression of its spread, it has now caused a pandemic.

This pandemic represents the most serious global public health threat since the devastating 1918 H1N1 influenza pandemic. Justifiably, several countries have adopted draconian measures to combat this threat. The scientific community, in addition to its accelerated efforts to develop an effective treatment and a vaccination, is also playing an important role in advising policymakers of possible non-pharmacological approaches to limit the catastrophic impact of the pandemic. For example, two possible strategies, called mitigation and suppression, are thoroughly discussed in the important paper [6]; in the early stages of the pandemic, the UK was following mitigation, but after the publication of this report, is now following suppression.

In this paper, we present a novel methodology for *predicting the time evolution of the cumulative number, N(t), of the individuals reported to be infected in a given country, by* SARS-CoV-2. This methodology can be used for predicting several features of the epidemic, such as the time that a plateau will be reached, as well as the total number of individuals reported to be infected at that time. Here, *the plateau is defined as the time when the rate of change of the people reported to be infected is 5% of the maximum rate of infection*.

Our methodology is based on two different tools: on the use of appropriate mathematical models and on the employment of deep learning networks. Regarding the first tool, our mathematical models have led to two analytical formulae, called *rational* and *birational*. The advantage of these formulae is that they provide more accurate predictions for the characteristics of the plateau than the classical logistic formula often used in epidemiology. Also, importantly, the birational model may provide an upper bound of *N*(*t*), and hence it is preferable to the rational one. However, the rational model can be constructed sooner than the birational one: the input needed for the rational model is data until around the time when the maximum rate of infection occurs, which will be denoted by T (the corresponding point on the curve describing *N*(*t*) is known as the *inflection point*). On the other hand, the birational model requires data for several more additional days. The effectiveness of the above two analytical formulae is supported by showing that their predictions are as accurate as those obtained via a deep learning algorithm; in particular, we employed a bidirectional long short-term memory (BiLSTM) network, which provides a powerful generalization of recurrent neural networks.

**Figure 1.**
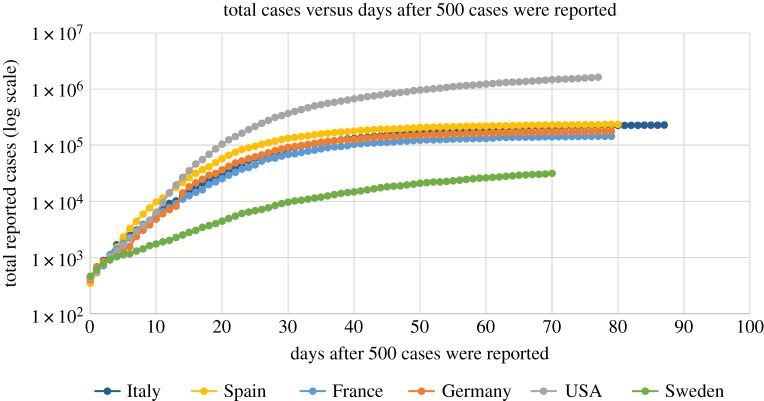
SARS-CoV-2 virus epidemics in Italy, Spain, France, Germany, USA and Sweden: total cumulative number of individuals reported to be infected up to 24 May 2020, as a function of days after the day that 500 cases were reported.

**Figure 2.**
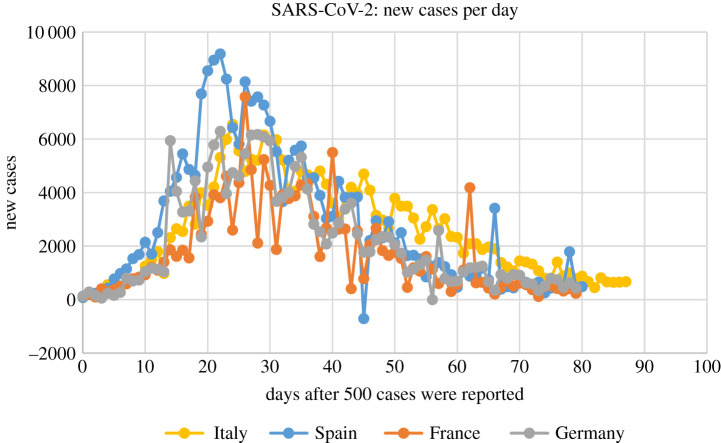
SARS-CoV-2 virus epidemics in Italy, Spain, France and Germany: new reported cases up to 24 May 2020, as a function of days after the day that 500 cases were reported.

A prerequisite for the development of any accurate model is the existence of appropriate data. For the pandemic of SARS-CoV-2 such data are certainly available. For example, there exist a long series of data from Italy, Spain, France and Germany, where their SARS-CoV-2 epidemics are approaching or have passed the plateau; USA and Sweden passed the inflection point several weeks ago but appear to have a slow approach towards a plateau. The total reported cases of the above countries as a function of the number of days after the day that 500 cases were reported are shown in figure 1. Estimated rates of change (new reported cases) for Italy, Spain, France and Germany are plotted in figure 2.

These graphs show that in all countries, except Sweden, the growth of the epidemics is similar for the first approximately 10 days after the day that the number of infected persons reached 500. However, following this period, the behaviour of the epidemics is different, presumably reflecting the type of measures and the time that these measures were implemented, in each country.

The mathematical modelling of epidemics has a long and illustrious history; it began with the Kermack–McKendrick model, introduced in 1927 [7]. In this pioneering paper, the population is divided into susceptible, infectious and recovered (removed) sub-populations. Then, specific ordinary differential equations are formulated characterizing the time evolution of the functions representing these populations. The above work was certainly ahead of its time. It was rediscovered in the 1980s, and since then it has provided the basis for a variety of deterministic models, known as SIR models (rigorous mathematical results for such models are derived in [8]). The generalization of SIR to models involving partial differential equations is presented in [9]. A simple extension of the standard SIR model capable of modelling SARS-CoV-2 is presented in [10]. This model involves six ordinary differential equations (ODEs) specified by nine parameters. There is an underlying belief in the mathematical epidemiology community that these parameters, in principle, can be determined from the epidemiological data of the number of infected and diseased individuals. However, the analysis of the above six ODEs presented in [10] shows that this is *not possible* (on the other hand, it is possible to determine those *combinations* of the nine model parameters that specify a fourth-order ODE characterizing the time evolution of the number of deaths [10]).

In addition to the above formidable obstacle rigorously derived in [10] concerning the determination of the SIR model parameters, the number of the individuals infected by SARS-CoV-2 is obviously different than the number of individuals *reported* to be infected. These considerations make it necessary to seek a direct approach to modelling the accumulative number *N(t)* of individuals reported at time *t* to be infected by a viral epidemic. In this work, we *assume* that the function *N*(*t*) satisfies the ordinary differential equation

1.1 $$\frac{\text{d}N(t)}{dt}=\alpha (t)\left(N(t)-\frac{N{\left(t\right)}^{2}}{N_{f}}\right).$$

This is a Riccati equation that is specified by the time-dependent function *α*(*t*) and the constant parameter *N_f_.* This constant as well as the function *α*(*t*) depend on the basic characteristics of the particular virus and on the cumulative effect of the variety of different measures taken by the given country for the prevention of the spread of the viral infection. The dependence of *α*(*t*) on time reflects various time-dependent factors, including the fact that the effect of the different measures taken by the government depends on *t*. The case of *α(t)=constant* can be considered as an ‘ideal’ case.

Remarkably, although (1.1) is a nonlinear equation depending on time-dependent coefficients, it can be solved in closed form. Its solution depends on *α*(*t*), the constant parameter *N_f_*, and the constant of integration *β*:

1.2 $$N=\frac{N_{f}}{1+\beta {\text{e}}^{-\tau }}\;,\tau =\int \alpha (t)\text{d}t\;.\;$$

In the particular case that *α*(*t*) is a constant denoted by *κ*, equations (1.2) yield the classical logistic formula

1.3 $$N=\frac{N_{f}}{1+\beta {\text{e}}^{-\kappa t}}\;.$$

**Figure 3.**
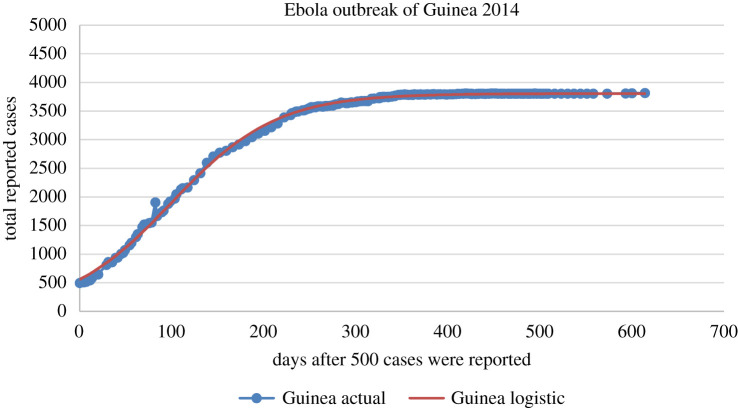
Ebola outbreak of Guinea in 2014: predicted versus actual for the total cumulative number of individuals reported to be infected with the Ebola virus, as a function of days after the day that 500 cases were reported. The logistic formula given by equation (1.3) provides an excellent fit for the actual data.

Interestingly, this simple formula is adequate for capturing the evolution *N*(*t*) of typical viral epidemics. For example, determining the three constant parameters *κ*, *β* and *N_f_* of equation (1.3) with data from the Ebola virus epidemic of 2014 in Guinea, we find the excellent fit depicted in figure 3. Importantly, the above parameters remain essentially unchanged if we use a *smaller set* of data for their determination, which shows that the logistic model could also have been used for *predictive* purposes (throughout this paper the unknown parameters are determined by employing an error-minimizing algorithm described in §2.1). The cases of Liberia and Sierra Leone are very similar to the case of Guinea (the relevant data were obtained from the official site of the Centers for Disease Control and Prevention (CDC); they are official World Health Organization (WHO) data)^1^.

**Figure 4.**
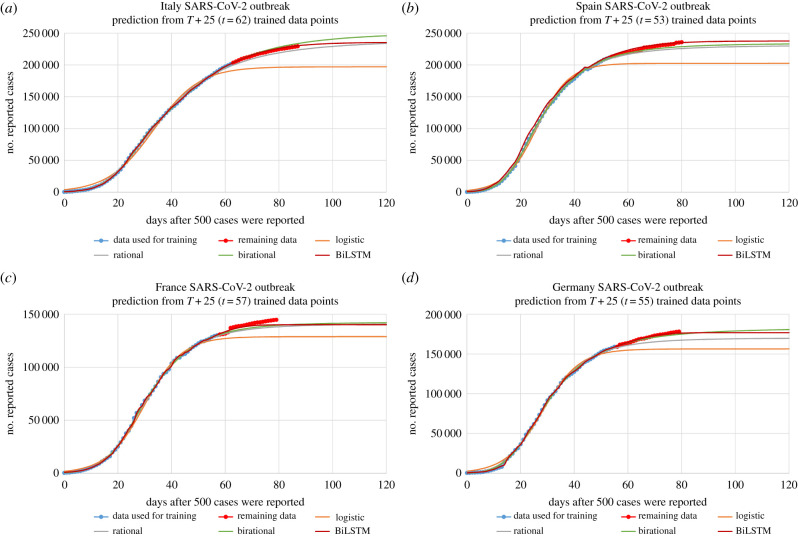
Predictions using a smaller training dataset for the cumulative number of reported cases as a function of days after 500 cases were reported for: (*a*) Italy, (*b*) Spain, (*c*) France and (*d*) Germany. The prediction fits were obtained using training data up to *T* + 25 for each country, which for Italy, Spain, France and Germany, corresponds to *t* = 62 (*T* = 37), *t* = 53 (*T* = 28), *t* = 57 (*T* = 32) and *t* = 55 (*T* = 30), respectively. The inflection point T in ‘*T* + 25’ was calculated by training the logistic function with data up to 24 May 2020. The models were trained with the data shown in light blue, and then were used to predict the remaining data shown in red.

As it will be shown in §3, the simple formula (1.3) also provides a good fit of the SARS-CoV-2 pandemic. However, the long series of existing data of the epidemics of Italy, Spain, France and Germany show that the logistic model does *not* provide accurate predictions. For example, figure 4*a* shows that if we use a subset of the existing data of the epidemic in Italy for the determination of the parameters of the logistic model, and then compare the resulting graph of *N*(*t*) with the remaining available data, we find that the logistic model *underestimates N(t)*; in other words, the logistic model provides a *lower bound* of the actual *N(t)*. This raises the following natural question: is it possible to find a formula yielding more accurate predictions than the logistic one? After experimenting with more than 50 different forms of *α*(*t*), we have obtained an affirmative answer to the above question, by introducing two novel formulae which will be referred to as *rational* and *birational*. In the former case, the exponential function appearing in equation (1.3) is replaced by an algebraic function; in the birational formula, the values of the parameters specifying this algebraic function change, depending on whether *t* is larger or smaller than a parameter denoted by *X*.

In this work, we implemented the following tasks associated with the epidemics in Italy, Spain, France and Germany: (i) we determined the parameters of the logistic, rational and birational formulae, by training the above formulae using only a subset of the data, namely data up to *T* + 25 (the inflection point *T* was determined by fitting the logistic model over the whole dataset of each country, namely up to 24 May 2020). By plotting the prediction curves of these formulae against the actual data up to 24 May 2020, it becomes evident that first, the logistic formula provides a lower bound, and second, that the rational and birational formulae generate more accurate predictions. Furthermore, for the cases of Italy, the birational formula generates a curve which is *above* the curve of the data, suggesting that the birational model may provide an upper bound. (ii) We established that the rational and birational formulae have similar predicting performance with a far more complicated deep learning network, which does not depend on the assumption of the validity of equation (1.1). This finding highlights the suitability of the proposed mathematical models for predictive purposes. (iii) We computed the time of the plateau as well as the value of *N* at the plateau using the logistic, the rational and the birational formulae, as well as the BiLSTM network.

In particular, the birational model yielded the following estimates for the dates that the plateau will be reached, as well as for the number of individuals reported to be infected with SARS-CoV-2 when this occurs: Italy, plateau on 11 June 2020, with 242 315 individuals reported to be infected (as noted above, the estimate for Italy may be an upper bound); Spain, plateau on 17 May 2020, with 226 573 reported individuals; France, plateau on 21 May 2020, with 138 873 reported individuals; Germany, plateau on 23 May 2020, with 174 356 individuals.

According to the distinguished philosopher of science Karl Popper, a necessary requirement for the validity of a theory is that it is falsifiable. This notion suggests that supportive evidence for the validity of our approach can be provided by presenting cases where our models fail. For this purpose, we have investigated the epidemics of Sweden and of USA. In the case of Sweden, only partial restrictions were applied instead of strict lockdown measures, whereas the different measures adopted by different states makes USA very difficult to model. Indeed, for the epidemics in these two countries, after using a subset of the available data to train the three models and the neural network, we could not obtain curves that were close to the curves of the remaining data. Thus, for USA and Sweden, we have presented the parameters determined with data up to 24 May 2020, with the understanding that we do not expect neither the associated analytical formulae nor the BiLSTM network to provide accurate predictions (for these two countries the following numbers provide rather *inaccurate* lower bounds: USA, plateau on 28 August 2020, with 2 264 338 reported individuals; Sweden, plateau on 9 October 2020, with 57 540 reported individuals).

## 2. The basic model

Let *F* denote the *relative infectivity* of the epidemic, defined by

2.1 $$F=\frac{\text{d}N/dt}{N}\;.$$

We *assume* that *F* is a *linear*, *time-dependent function of N*. Let the constant *N_f_* denote the final cumulative number of individuals reported to be infected. Taking into consideration that *F* vanishes when *N = N_f_*, we have

2.2 $$F=\alpha (t)\left(1-\frac{\text{N}}{N_{f}}\right).$$

Inserting equation (2.2) in the definition (2.1) we find the basic equation modelling this situation, namely the Riccati equation (1.1).

The particular case of a Riccati equation with constant coefficients (corresponding to the case that *α*(*t*) is constant) has appeared in a plethora of dynamic processes, including the modelling of epidemics. Indeed, in the classical SIR model mentioned in the introduction, if one assumes that *R* = 0, then after replacing in the first-order differential equation satisfied by *I, S* with *I-T,* where the constant *T* denotes the total population, one finds a Riccati equation of the form (1.3), where *α*(*t*) is replaced by a *constant*. Another notable example of the appearance of a constant coefficients Riccati equation in the mathematical modelling of infectious processes can be found in the paper of Anderson and May [11]; this work describes the dynamic interaction of parasites with their host environment. In this paper, whose impact in the field of mathematical biology was far reaching [12], a Riccati equation is formulated that involves a *single constant* parameter^2^.

In order to solve the Riccati equation (1.1), we first use the independent change of variables specified by the second of equations (1.2). This gives rise to an equation similar to equation (1.1), where *α* is replaced by *1* and *t* by *τ*. This constant coefficients Riccati equation can be linearized via the change of the dependent variable specified by

2.3 $$N=\frac{\text{d}y/d\tau }{y}\;.$$

Indeed, substituting equation (2.3) into the above constant coefficients Riccati equation and simplifying, we find

$$\frac{{\text{d}}^{2}y}{{\text{d}}^{2}\tau }=\frac{\text{d}y}{d\tau }\;.$$

Solving this equation, substituting the resulting expression in equation (2.3), and simplifying, we find the first expression in equation (1.2).

We expect that the validity of the above model improves as *t* increases. Thus, we avoid evaluating the first of equations (1.2) at *τ =0* to express *β* in terms of *N_f_* and *N* at *τ* = 0. Instead we determine *β* by matching the expression obtained from the first of equations (1.2) with the actual data.

Important information provided by the above model is the time *T* when the maximum rate of infection is achieved: computing the second derivative of the right-hand side of the first of equations (1.2) and requiring that the resulting expression vanishes, we find that *T* satisfies the equation

2.4 $${\text{e}}^{\int_{0}^{T}a(t)\;dt}=\beta {\left[\frac{a^{2}+a^{\prime }}{a^{2}-a^{\prime }}\right]}_{t=T}\;,$$

where throughout this paper prime denoted differentiation with respect to time. Using the above expression in the exponential occurring in the expressions for *N* and *N*′ evaluated at *t = T*, we find.

2.5 $$\begin{array}{rl} N(T) & =N_{f}\left[\frac{1}{2}+{\left(\frac{a^{\prime }}{a}\right)}_{t=T}\right]\;,\\ N^{\prime }(T) & =\frac{N_{f}}{4}{\left[\frac{1}{a}\left(\frac{a^{2}-a^{\prime }}{a^{2}+a^{\prime }}\right)\right]}_{t=T}. \end{array}$$

For the logistic model we have *α*(*t*) = *κ*. Thus, equations (2.4) and (2.5) yield

2.6 $$T=\frac{\ln(\beta )}{k},\;\;\;\;\;N(T)=\;\frac{{\text{N}}_{f}}{2}\;,\;\;N^{\prime }(T)=\frac{N_{f}}{4k}.$$

Taking into consideration that the logistic formula is a good approximation of the relevant dynamic process, the above value of *T* provides an *approximate* value for the time that the inflection point is reached.

The rational and birational models are defined, respectively, as follows:

2.7 $$N=\frac{N_{f}}{1+b{\left(1+dt\right)}^{-k}}\;,$$

and

2.8 $$N=\left\{ \begin{array}{ll} \frac{c}{1+b{\left(1+dt\right)}^{-k}}, & t\leq X\\ \begin{array}{ll} & \frac{c}{1+b{\left(1+dX\right)}^{-k}}-\frac{c_{1}}{1+b_{1}{\left(1+d_{1}X\right)}^{-k_{1}}.}\\ & \;+\frac{c_{1}}{1+b_{1}{\left(1+d_{1}t\right)}^{-k_{1}}}, \end{array} & t>X \end{array}\right.,$$

where *X* is a constant parameter in the vicinity of *T*.

Letting in equation (2.8) t→∞, we find

2.9 $$N_{f}=\frac{c}{1+b{\left(1+dX\right)}^{-k}}-\frac{c_{1}}{1+b_{1}{\left(1+d_{1}X\right)}^{-k_{1}}}+c_{1}.\;$$

By comparing equations (2.7) and (2.8) with the first of equations (1.2), it is straightforward to determine *α(t)* for both the rational and the birational models: for the rational model

$$\alpha (t)=\frac{kd}{1+kt}\;,$$

which justifies the terminology ‘rational’. For the birational model

$$\alpha (t)=\{\begin{array}{ll} \frac{k\text{d}}{1+\text{d}t}, & t\leq X\\ \frac{k_{1}{\text{d}}_{1}}{1+{\text{d}}_{1}t}\frac{1}{1+(1-(c_{1}/N_{f})){\left(1+{\text{d}}_{1}t\right)}^{-k_{1}}}, & t>X. \end{array}$$

If *b*, *c, d, k* are close to *b*_1_, *c*_1,_ *d*_1_, *k*_1_, then *N_f_* is close to c_1,_ and hence the value of *α*(*t*) for *t > X* is close to the value of *α(t)* for *t* < *X*.

Computing the second derivative of the right-hand side of equation (2.7) and equating the resulting expression to zero, we find that the value of *T* for the rational model is characterized by the equation

2.10 $${\left(1+dT\right)}^{k}=b\left(\frac{k-1}{k+1}\right).\;$$

Similarly, for the birational model where the parameters *b*, *d*, *k* are replaced with *b*_1_, *d*_1_, *k*_1,_ respectively.

Replacing the rational function in the expressions for *N* and of its derivative with the rational function of equation (2.10), we find that for the rational model

2.11 $$N(T)=\;\frac{{\text{N}}_{f}}{2}\left(1-\frac{1}{k}\right),\;\;N^{\prime }(T)=N_{f}\frac{d}{4k}\frac{k^{2}-1}{1+dT}.$$

Similar expressions are valid for the birational model.

The birational model is based on the natural assumption that the parameters of the rational function specifying the function *N*(*t*) are different before and after *T*. It is quite satisfying that this very simple model yields the best fits among more than 50 models that were tested. Included in these models were several ‘fractal’ models; in the simplest such model, the exponent kt in the logistic formula was replaced with *k* × *t^μ^*. Among other models investigated was the generalized logistic formula

$$N(t)=N_{l}+\frac{N_{u}-N_{l}}{1+{\text{e}}^{-\kappa (t-T)}}\;,$$

where *N_l_* and *N_u_* are the upper and lower values of the relevant curve. This expression provides the general solution of the time-independent ODE

$$\frac{\text{d}N}{\text{d}t}=\frac{k}{N_{u}-N_{l}}(N-N_{l})(N_{u}-N).\;$$

It turns out that this equation is actually equivalent with the time-dependent ODE (1.1), where

$$N_{f}=N_{u},\;\alpha (t)=\;\frac{k}{1+(N_{l}/N_{u}){\text{e}}^{-k(t-T)}}.$$

In other words, the generalized logistic formula is the general solution of equation (1.1) with the above choices of *N_f_* and α(*t*).

### 2.1. Optimization method

We obtained the time-series data for the coronavirus disease (COVID-19) for the counties studies here from the official site of the European Centre for Disease Prevention and Control.^3^ We arranged the data in the form of individuals *N* reported to be infected over time measured in days, after the day that the number of cases reached 500.

All evaluated formulae were fitted using the *simplex algorithm*, which is an iterative procedure that does not need information regarding the derivative of the function under consideration. The algorithm creates a ‘random’ simplex of *n* + 1 points, where *n* is the number of the model parameters that need to be estimated. The simplex changes iteratively by reflection, expansion and contraction steps until it finds the model parameters that minimize the given likelihood function. The constrained variation of the simplex algorithm [13,14] available in MATLAB^®^ was used for all tested formulae; an L_1_*-norm* was employed in the likelihood function to improve robustness [15]. The simplex algorithm is particularly effective for cases where the gradient of the likelihood functions is not easy to calculate. Random parameter initializations were used to avoid local minima. The simplex algorithm was chosen because it performed better than certain nonlinear least-squares curve fitting algorithms evaluated in this work, namely the Levenberg–Marquardt [16] and the trust-region-reflective [17] algorithms.

The stability of the fitting procedure was established using the following simple criterion: different fitting attempts based on the use of a fixed number of data points must yield curves which have the same form *beyond* the above fixed number of points. The fitting accuracy of each model was evaluated by fitting the associated formula on *all* the available data in a specified set. The relevant parameters specifying the logistic, rational and birational formula are given in table 1. For computing the inflection point *T*, we require the time that the derivative of *N* becomes maximum. For this purpose, we used the model with the best fit in the neighbourhood of the inflection point, which in most cases turned out to be the birational model. For all countries presented for the fitting of the birational model we used *X = T*.

**Table 1.** Model parameters and plateau characteristics for the logistic, rational, birational models as well as for the BiLSTM network, for the SARS-CoV-2 epidemics of Italy, Spain, France and Germany. For completeness, the epidemics of USA and Sweden are also included, although for these cases all three analytical formulae and the deep learning network are not able to provide accurate predictions.

	Italy	Spain	France	Germany	USA	Sweden
logistic model						
*N_f_*	197 140	202 595	128 894	156 381	1 596 647	36 289
*k*	0.1176	0.1663	0.1420	0.1450	0.0899	0.0721
*β*	50.7112	76.6129	70.2454	59.7248	56.9872	26.3107
*T*	33	26	30	28	45	45
plateau (days)	70	54	62	58	95	107
plateau (cases)	194 511	200 661	127 544	154 334	1 579 077	35 868
*R*^2^	0.9952	0.9965	0.9981	0.9969	0.9935	0.9968
*RMSE*	4999	4689	2112	3312	45 299	602
rational model						
*N_f_*	240 309	230 987	141 032	170 124	2 143 854	56 472
*k*	3.1823	3.9391	5.3226	5.3035	3.0843	2.7294
*b*	6257.6849	1999.5016	433.2972	399.9912	3079.8451	183.0564
*d*	0.39307	0.21195	0.06882	0.07179	0.23336	0.09069
plateau (days)	101	72	75	68	157	210
plateau (cases)	229 472	223 414	137 306	164 659	2 056 370	53 724
*R^2^*	0.9994	0.9996	0.9996	0.9994	0.9988	0.9996
*RMSE*	1761	1583	940	1499	19 331	214
birational model						
*c*	158 859	191 523	142 712	180 587	1 588 919	38 949
*k*	8.9480	6.6694	7.1566	4.5688	3.8026	4.1501
*b*	285.1039	542.1584	277.3544	871.7813	4013.6717	87.6494
*d*	0.03068	0.06346	0.03896	0.11310	0.17924	0.04060
*c_1_*	221 318	224 369	126 839	206 283	2 113 840	58 016
*k*_1_	4.5998	4.4835	6.1164	3.4905	3.1932	3.6924
*b*_1_	142.7772	287.9202	297.1107	445.5426	2899.4413	57.7578
*d_1_*	0.04595	0.08890	0.04669	0.16854	0.17723	0.02931
plateau (days)	105	73	76	78	173	211
plateau (cases)	242 315	226 573	138 873	174 356	2 264 338	57 540
*R^2^*	0.9999	0.9998	0.9997	0.9997	0.9996	0.9997
*RMSE*	538	1022	821	1128	10 754	178
BiLSTM Network						
plateau (days)	92	75	71	75	125	105
plateau (cases)	232 072	233 641	139 383	175 357	2 072 246	46 111
*R*^2^	0.9999	0.9993	0.9993	0.9998	0.9997	0.9999
*RMSE*	764	4069	1578	807	23 397	490

## 3. Deep learning

Machine learning and in particular deep learning have had a transformative impact in many areas of science and technology; furthermore, they have begun to have a significant impact in medicine [18]. The first important relevant applications of neural networks were related to the employment of artificial neural networks (ANN) with one hidden layer and a finite number of neurons. According to the universal approximation theorem [19], by estimating the weights of these neurons, ANN can learn any nonlinear function; but this may require a large number of neurons. In order to fit complex multivariate functions, such as those required to model the number of individuals reported to be infected by SARS-CoV-2, deeper neural networks (many hidden layers) may provide a more efficient alternative. Each hidden layer of an ANN has its own weights and acts independently, hence ANN cannot capture sequential information of time series, such as the cumulative number of individuals reported to be infected. On the other hand, this can be easily achieved using recurrent neural networks (RNN), where the current state of the hidden layers *h_t_* at each time step *t* is estimated via the process of combining the current input *x_t_* with the previous state of the hidden layers *h*_*t*−1_:

$$h_{t}=\sigma f(w_{hh}h_{t-1}+w_{xh}x_{t}).$$

In this equation, *σ* is the activation function, *w_hh_* are the weights of the recurrent neurons, and *w_xh_* are the weights of the input neurons. The final state of the hidden layers is estimated after the network goes, sequentially, through all the inputs of the time series. The prediction of the RNN is given by $y_{t}=w_{hy}h_{t}$, where *w_hy_* are the weights of the output neurons.

A common problem with both ANN and RNN, particularly when many hidden layers are used, is the vanishing or the exploding of the gradient in the backpropagation algorithm occurring during the process of updating the weights. Unlike ANN, RNN can capture sequential information, but cannot learn from long-term dependencies. Hochreiter & Schmidhuber introduced in [20] the long short-term memory (LSTM) networks that can capture long-term dependencies and at the same time avoid the problem of vanishing/exploding gradients. LSTM are a type of RNN, where a memory cell maintains its state over time; they use gates to decide whether to flow information in (keep) or out (forget) of the memory cell. The first step of LSTM decides which information to ‘forget’ from a memory cell state; this step and can be expressed in the form

$$f_{t}=\sigma (w_{hh}^{f}h_{t-1}+w_{hx}^{f}x_{t}+b_{f}),$$

where *σ* is an activation function (usually sigmoid) to output a value$\;f_{t}\in [0,1]$_._ For ***f_t_*** = 1, it flows all information in the memory cell *C*_*t*−1_, whereas for *f_t_* = 0 it forgets all information. The second step involves the input gates, denoted by *in_t_* and *Ĉ_t_*, respectively, which synergistically decide which information will be added in the memory cell. First, the input is restricted between −1 and 1 using the *tanh* layer, ${\hat{C}}_{t}=\text{tan}h(w_{hh}^{C}h_{t-1}+w_{hx}^{C}x_{t}+b_{C})$. Then, *Ĉ_t_* is multiplied by the input gate *in_t_* to decide the values to be added in the current state of the memory cell to replace the ones the network forgot:

$$in_{t}=\sigma (w_{hh}^{in}h_{t-1}+w_{hx}^{in}x_{t}+b_{in}).$$

The current state C_t_ aggregates the old memory state via the forget gate, $C_{t}=C_{t-1}\;*\;f_{t}+{\hat{C}}_{t}\;*\;in_{t}$. In the final step, the output gate decides which information of the cell state to output:

$$\text{ou}{\text{t}}_{t}=\sigma (w_{hh}^{\mathrm{out}}h_{t-1}+w_{hx}^{\mathrm{out}}x_{t}+b_{\mathrm{out}}).$$

The final output of the cell memory is given by $h_{t}=\tan h(C_{t})\;*\;\text{ou}{\text{t}}_{t}$.

Graves & Schmidhuber introduced in [21] the BiLSTM networks; this development was motivated by the bidirectional RNN networks, introduced earlier by Schuster & Paliwal in [22], where the training runs forwards and backwards using two separate RNN. Consequently, the main difference is the training sequence: in the LSTM, the sequence runs backwards, preserving information from the future, whereas in the bidirectional LSTM training, the sequence runs backward and forward preserving information from both the past and the future. The resulting forward and backward hidden values of the bidirectional LSTM, namely *h^f^* and *h^b^* respectively, are concatenated giving the final output *h_t_* = (*h^f^*,*h^b^*). The bidirectional LSTM are well suited for time-series prediction and can potentially completely capture the contextual information of the time series.

### 3.1. Implementation

Several machine algorithms have been validated for the purposes of this study such as ANN, RNN, LSTM and BiLSTM. The BiLSTM network was chosen based on fitting performance and prediction accuracy. The relevant model was implemented in Matlab; it consisted of two Bi-LSTM layers and a fully connected layer, using the ReLu activation function. The model was optimized by the algorithm of adaptive learning rate [23], using as a loss function the root mean square error (plus an L_2_ regularization term to avoid overfitting) at learning rate 0.01. The batch size used was 1. Dropout was included in the bidirectional LSTM layer, and the proportion of disconnection was 0.1. Each model was optimized by training for 2000 epochs. Hyperparameters such as the number of hidden units and the regularization factor of the loss function were optimized using, as part of the training, a grid search approach for each network.

## 4. Results

Table 1 presents the parameters for the three different models as applied to Italy, Spain, France, Germany, USA and Sweden. This table also presents the fitting accuracy and plateau characteristics for all the aforementioned countries for all three mathematical models, as well as for the BiLSTM prediction. For Italy, Spain, France and Germany, only a subset of the available data were trained, namely data up to *T* + 25. In this case, T corresponds to the inflection point of the complete dataset, namely data up to 24 May 2020. The constant *T*, where the inflection point occurs, was determined by fitting the logistic model in the complete dataset (the other models yield similar results). For Italy, Spain, France and Germany, the inflection point occurred at *t* = 37, *t* = 28, *t* = 32 and *t* = 30, respectively. The inflection point for USA and Sweden, which was also determined from the complete set of data (up to 24 May 2020), occurred at *t* = 45 for both countries; for USA and Sweden this corresponds to 22 and 26 April 2020, respectively.

Incidentally, if one uses data only up to T + 25, then the inflection point is slightly different than the one computed from all the data (up to 24 May 2020): for Italy, Spain, France and Germany, it is found to occur at *t* = 33, *t* = 26, *t* = 30 and *t* = 28, respectively (table 1). This corresponds to 31 March, 31 March, 5 April and 3 April 2020, respectively.

Figure 4 presents the predictions made by the analytical formulae and the deep learning network versus the actual data for the cumulative number of reported cases due to SARS-CoV-2, as a function of days after 500 cases were reported, for the epidemics in Italy, Spain, France and Germany. The parameters of the analytical formulae and for the deep learning network were obtained using a smaller set of the available data for each country, namely up to *T* + 25, which for Italy, Spain, France and Germany, corresponds to *t* = 62, *t* = 53, *t* = 57 and *t* = 55, respectively. It is important to emphasize that each of the three mathematical models, namely equations (1.3), (2.7), and (2.8), as well as the deep learning model, can fit the trained data quite well (see R^2^ in table 1). However, the predictive capacity of these formulae is *not* the same. This is best illustrated by comparing the predictive curves of each model with the remaining available data up to 24 May 2020 (in red). For the epidemic of Italy (figure 4*a*), the logistic model predicts a plateau on 7 May 2020 (70 days after the day that 500 cases were reported) with 194 511 individuals reported to be infected by SARS-CoV-2; the rational model predicts a plateau on 7 June 2020 (day 101) with 229 472 reported individuals; the birational model predicts a plateau on 11 June 2020 (day 105) with 242 315 reported individuals and the BiLSTM network predicts a plateau on 29 May 2020 (day 92) with 232 072 reported individuals. Clearly, the logistic model already underestimates the actual plateau day and the number of reported cases, since on 24 May 2020 (last day of acquired data for this study) the number of reported cases for Italy had reached 229 327.

For the epidemic of Spain (figure 4*b*), the logistic model predicts a plateau on 28 April 2020 (day 54 after the day that 500 cases were reported) with 200 661 reported individuals; the rational model predicts a plateau on 16 May 2020 (day 72) with 223 414 reported individuals; the birational model predicts a plateau on 17 May 2020 (day 73) with 226 573 reported individuals and the BiLSTM network predicts a plateau on 19 May 2020 (day 75) with 233 641 reported individuals. Again, the logistic model clearly underestimates the actual plateau day and the number of reported cases, since on 24 May 2020, the number of reported cases for Spain had reached 235 772.

For the epidemic of France (figure 4*c*), the logistic model predicts a plateau on 7 May 2020 (day 62 after the day that 500 cases were reported) with 127 544 reported cases; the rational model predicts a plateau on 20 May 2020 (day 75) with 137 306 reported cases; the birational model predicts a plateau on 21 May 2020 (day 76) with 138 873 reported cases and the BiLSTM network predicts a plateau on 16 May 2020 (day 71) with 139 383 reported cases. Again, the logistic model clearly underestimates the actual plateau day and the number of reported cases, since on 24 May 2020, the number of reported cases for France had reached 144 806.

For the epidemic of Germany (figure 4*d*), the logistic model predicts a plateau on 3 May 2020 (day 58 after the day that 500 cases were reported) with 154 334 reported cases; the rational model predicts a plateau on 13 May 2020 (day 68) with 164 659 reported cases; the birational model predicts a plateau on 23 May 2020 (day 78) with 174 356 reported cases and the BiLSTM network predicts a plateau on 20 May 2020 (day 75) with 175 357 reported cases. Again, the logistic model clearly underestimates the actual plateau day and the number of reported cases, since on 24 May 2020, the number of reported cases for Germany had reached 178 281.

For completeness, we also present the time of the plateau and the corresponding value of N for the epidemics of USA and Sweden, although, as stated in the introduction, neither the explicit formulae nor the deep learning network provide accurate predictions. As stated earlier, in these cases, the three explicit formulae and the BiLSTM network were trained with data up to 24 May 2020. For the epidemic of USA the logistic model predicts a plateau on 11 June 2020 (day 95 after the day that 500 cases were reported) with 1 579 077 reported cases; the rational model predicts a plateau on 12 August 2020 (day 157) with 2,056,370 reported cases; the birational model predicts a plateau on 28 August 2020 (day 173) with 2 264 338 reported cases and the BiLSTM network predicts a plateau on 11 July 2020 (day 125) with 2 072 246 reported cases.

For the epidemic of Sweden, the logistic model predicts a plateau on 27 June 2020 (day 107 after the day that 500 cases were reported) with 35 868 reported cases; the rational model predicts a plateau on 8 October 2020 (day 210) with 53 724 reported cases; the birational model predicts a plateau on 9 October 2020 (day 211) with 57 540 reported cases and the BiLSTM network predicts a plateau on 25 June 2020 (day 105) with 46 111 reported cases.

## 5. Conclusion

Several useful models elucidating aspect of the COVID-19 pandemic have already appeared in the literature; they include the following: (i) a model for simulating the transmissibility of SARS-CoV-2 from bats to humans is presented in [24]. (ii) The calculations of exponential growth and maximum likelihood are used in [25] to determine the reproductive number of SARS-CoV-2 and SARS in China. (iii) The formulation of a susceptible–infected–recovered–dead (SIDR) model, together with the knowledge of data from China in the period 11 January to 10 February 2020, is used in [26] to estimate the associated per day infection mortality and recovery rates. (iv) In [27], by combining a stochastic model for the SARS-CoV-2 infection with the knowledge of data from China during January and February 2020, the probability that newly introduced cases might generate new outbreaks is calculated. (v) In [28], an SIDR model supplemented with mean-field kinetics is used to calculate the time and peak of confirmed infected individuals in China, Italy and France. (vi) In [29], the effect of social distancing was studied by using a model where the population was divided into those who are asymptomatic or have mild symptoms (95.6%), those who are hospitalized but do not require critical care (3.08%), and individuals who require critical care (1.32%); seasonal variations were incorporated by allowing the basic reproduction number to be a time-dependent function following a cosine curve that peaks in early December.

The above references, as well as the references [30–39] that also contain interesting results, represent only a tiny fraction of more than 3000 publications that have appeared in the last four months in arXiv, medRxiv and bioRxiv.

Here, we have modelled the cumulative number *N*(*t*) of persons reported to be infected by SARS-CoV-2 in a given country as a function of time, in terms of the Riccati equation (1.1). In addition, we have introduced a particular deep learning network which is capable of predicting accurately the time evolution of *N*.

Regarding equation (1.1), it is noted that although it is a nonlinear ODE containing time-dependent coefficients, it was solved in closed form, yielding (1.2). For appropriately chosen functions *α(t)*, the first of equations (1.2) provides a flexible generalization of the classical logistic formula that has been employed in a great variety of applications, including the modelling of infectious processes. The fact that *α* is now a function of *t* has important implications. In particular, it made it possible to construct the rational and birational formulae which provide more accurate predictions than the logistic formula.

**Figure 5.**
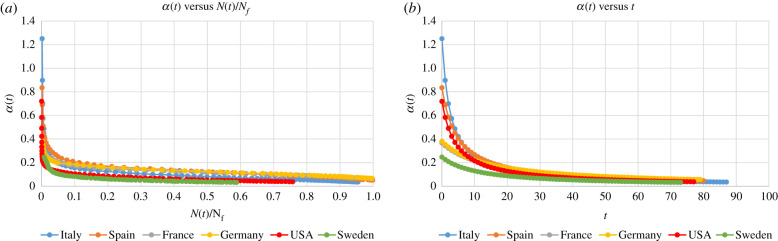
Plot of *α*(t) for the SARS-CoV-2 virus infection for Italy, Spain, France, Germany, USA and Sweden as a function of: (*a*) *N*(*t*)/*N_f_* and (*b*) *t*. After *t* around T-7, *α*(*t*) for all of the above countries is the same linear function of *N*(*t*).

The construction of the exact solution of equation (1.1), given by equations (1.2), has the following consequences: (i) for the case that an infection that has been stabilized, any of the three analytical formulae presented here can be used for the characterisation of the evolution of the cumulative number of persons reported to be infected. These expressions can be used for a variety of purposes. (ii) More importantly, the rational and birational models can be used for *predictive* purposes, providing accurate estimates for the characteristics of the plateau. (iii) Our approach has the capacity to provide increasingly accurate predictions: as soon as the epidemic in a given country passes the time *T*, the rational model can be used; furthermore, when the sigmoidal part of the curve is approached, the rational model can be supplemented with the birational model (a simple criterion of checking whether the birational model can be used is given in §2.1). Also, as more data become available, the parameters of the rational and of the birational models can be re-evaluated; this will yield better predictions. (iv) The Riccati equation (1.1) together with the flexibility of the arbitrariness of *α(t)*, offer the possibility of deciphering basic physiological mechanisms dictating the evolution of *N(t)*. In particular, following the transient stage of the epidemic, it is envisioned that *α(t)* becomes a function of *N* instead of a function of *t*. By plotting *α* in terms of *N* it is possible to scrutinize *a posteriori* such a relationship: we find that after *t* approximately equal to *T* − 7 the relation between *α* and *N/N_f_* is, remarkably, *linear*, *and almost identical for all studied countries*, figure 5.

How can the success of the simple mathematical model expressed by (1.1) be explained? Apparently, the constant *N_f_* defining equation (1.1), the constant of integration *β* entering the associated solution, and the constant parameters specifying the function *α*(*t*), capture the essence of the underlying time evolution process. This suggests that the cumulative effects of a variety of different mechanisms express themselves via the few parameters entering in the explicit solution formulae (1.3), (2.7) and (2.8). In this connection, it is worth recalling that the *single* parameter characterizing the Riccati equation of the celebrated Anderson–May model mentioned earlier represents the *cumulative effect* of different biological mechanisms^4^.

An additional partial explanation of the success of equation (1.1) is that the implementation of the formulae obtained via (1.1) for predicting the number of individuals infected by SARS-CoV-2 shares the same philosophy employed by the powerful technique of machine learning. Indeed, the explicit formulae (1.3), (2.7) and (2.8) used in this work can be thought of as ‘algorithms’, where given *t*, they predict *N*; these algorithms are characterized by several parameters, which are fixed by the ‘knowledge’ of the data. Thus, the more data are available, the better this algorithm ‘learns’ how to make accurate predictions. Hence, choosing these parameters by requiring that the analytical solution matches the data curve is consistent with the approach of machine learning.

An important part of this work is the presentation of detailed comparisons between the predictions of the analytical formulae and those obtained via a BiLSTM network. As discussed in the results section, the rational and birational formulae yield similar predictions with those of the above network.

As noted in the introduction, it is *not* possible using the epidemiological data of infected and deceased individuals to determine the parameters of SIR type models that specify the time evolution of the number of individuals infected by a given viral infection. On the other hand, *assuming that the number of individuals reported to be infected is a time-invariant percentage of the actual number of infected persons*, the analytical formulae as well as the deep learning algorithm discussed here *can* be used to predict the time evolution of the number of infected individuals. This information can be useful for a variety of purposes. In particular, following the expected decline of the ‘first wave’ of infections, several countries have begun easing the lockdown measures. This was vital, not only for economical but also for health considerations. Indeed, the psychological impact on the population at large of the current situation is substantial [40]; furthermore, this is expected to worsen, especially due to the effect of the post-traumatic disorder. Under the *assumption* that the characteristics of the virus remains unchanged, SIR type models predict that in the post-lockdown period the number of reported infected individuals as well as the number of deaths will begin to grow [10,41,42]. At this stage, the predictions made here and in [43] will cease to be accurate. However, these works are still very valuable: they can be used to compute the additional number of reported infected individuals and deaths caused by easing the lockdown measures. If these numbers are very small, it would mean that the assumption made in the SIR type models regarding the time invariance of the virus characteristic is no longer valid, and that *the virus has mutated to one which is less contagious and less virulent.* In this connection, we note that France begun easing the lockdown restriction on 11 May thus, since this effect will not be evident for approximately two weeks, it would not have been detected by 24 May 2020, which was the last day of our analysis. However, Germany begun easing the measures on 15 April (*t* = 40) and accelerated further this process on 30 April (*t* = 55) when, for example, playgrounds and churches were reopened; furthermore, all state-wide curfews were lifted on 9 May (*t* = 64). The fact that for the epidemic in Germany, our model predicts the situation quite well until at least 24 May, implies that the partial easing of the measures did *not* have any impact on the number of reported infected. This is most encouraging, pointing towards the possibility of the emergence of a less contagious virus.

We conclude with some general remarks: (i) taking into consideration the ubiquitous use of the logistic formula, and the fact that equations (2.7) and (2.8) provide variations of this formula, the rational and birational formulae may be useful for the modelling of a variety of phenomena. (ii) The fact that two different viral infections, namely SARS-CoV-2 and Ebola, are modelled by the same ODE, suggests that the Riccati equation (1.1) analysed here may play a generic role in the modelling of aspects of viral epidemics. (iii) The celebrated Burgers' equation, which is an evolution partial differential equation combining the generic effects of diffusion and nonlinear convection, admits a travelling wave solution that satisfies the Riccati equation (1.1) with *α*(*t*) a constant (this constant specifies the speed of propagation of the travelling wave, whereas *N_f_* is a free parameter appearing in Burgers' equation). Hence, the mathematical analysis presented in this work may also be relevant for some of the phenomena modelled by appropriate generalizations of the Burgers' equation.

## Supplementary Material

Updated prediction graphs

## Supplementary Material

Mathematical model results

## Endnotes

1 https://www.cdc.gov/vhf/ebola/history/2014-2016-outbreak/case-counts.html.

2 It should be noted that certain optimal features of the dynamics of the SIR model are also characterized by a Riccati equation that involves three time-dependent functions [12].

3 https://www.ecdc.europa.eu/en/geographical-distribution-2019-ncov-cases.

4 It is shown in [11] that this constant can be expressed in terms of the population size parameter *H*, the mortality rate of uninfected hosts parameter *b*, the mortality rate of infected hosts parameter *α* + *b*, and the rate at which infected hosts recover and become susceptible parameter *γ*.

## Data accessibility

The time-series data for the coronavirus disease (COVID-19) for the counties studies here were obtained from the official site of the European Centre for Disease Prevention and Control (https://www.ecdc.europa.eu/en/publications-data/download-todays-data-geographic-distribution-covid-19-cases-worldwide). Analytic spread sheets of our fitting results are available as part of the electronic supplementary material.

## Note added in proof

Figure 1 of the electronic supplementary material presents the updated curves of figures 4 for the period 24 May to 17 June. The curves associated with the epidemics of Italy and Spain, where most of the lockdown measures remain in effect, provide strong evidence for the capacity of our models to make accurate predictions. The curves associated with the epidemics of France and Germany provide strong evidence that the effect of the elimination of the lockdown measures has only a slight effect on the numbers of reported infected individuals as compared with the numbers predicted under the lockdown conditions. Remarkably, the large bump on the actual data of France occurred on 29 May (*t* = 84), which is indeed approximately two weeks after the easing of the lockdown measures (that occurred on 11 May). Furthermore, for the epidemic in Germany, the predicted curve began to deviate from the curve of the actual data on 25 May (*t* = 80), i.e. approximately two weeks after all state-wise curfews were eliminated.

A similar analysis presented in [43] shows that the number of deaths caused by COVID-19 has not been affected by the easing of the lockdown measures in the above countries. Taken together, *these results suggest that the virus responsible for the current pandemic, at least in the above four countries, has muted to one which is both less contagious and less virulent.* An alternative explanation is that the older and the vulnerable individuals continue to observe the lockdown measures.

For completeness, in figure 2 in the electronic supplementary material, we present a comparison of the actual data with predictions for the epidemics in USA and Sweden. These curves illustrate clearly the failure of both the analytical formulae and of the deep learning network to make accurate predictions.
