# Supplementary material for: Mathematical models and deep learning for predicting the number of individuals reported to be infected with SARS-CoV-2
Source: J R Soc Interface. 2020 Aug 5;17(169):20200494. doi: 10.1098/rsif.2020.0494 (PMC7482569; doi:10.1098/rsif.2020.0494)
Supplement: Updated prediction graphs [file rsif20200494supp1.docx]

**Supplementary material from “Mathematical models and Deep Learning for predicting the number of individuals reported to be infected with SARS-CoV-2” (updated on June 17, 2020)**


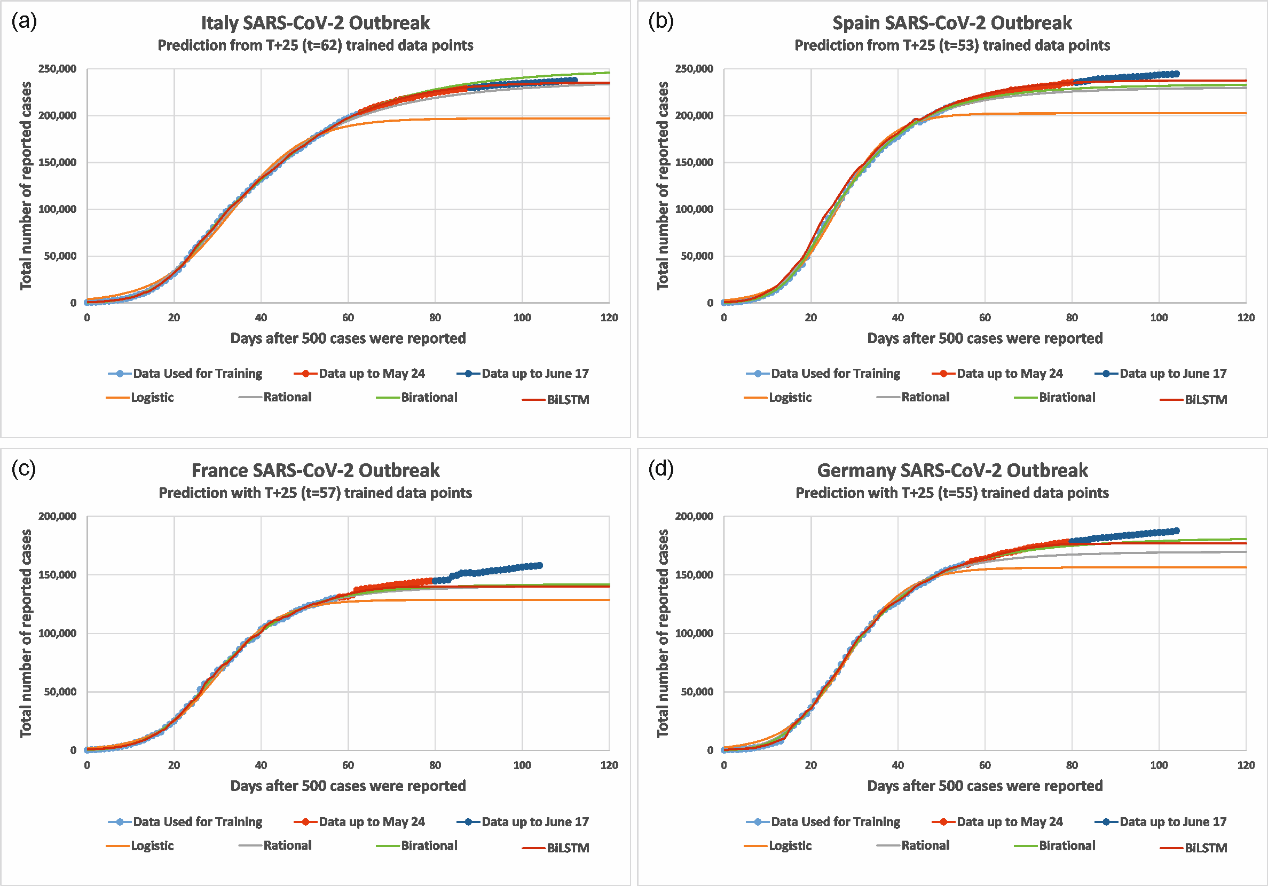


**Figure 1.** Predictions using a smaller training data set for the cumulative number of reported infected individuals as a function of days after 500 cases were reported for: **(a)** Italy, **(b)** Spain, **(c)** France, and **(d)** Germany. The prediction fits were obtained using training data up to T+25 for each country, which for Italy, Spain, France, and Germany, corresponds to t=62 (T=37), t=53 (T=28), t=57 (T=32), and t=55 (T=30), respectively. The models were trained with the data shown in light blue, and then were used to predict the data up to May 24 (shown in red) and the data reported after May 25 up to June 17, 2020.


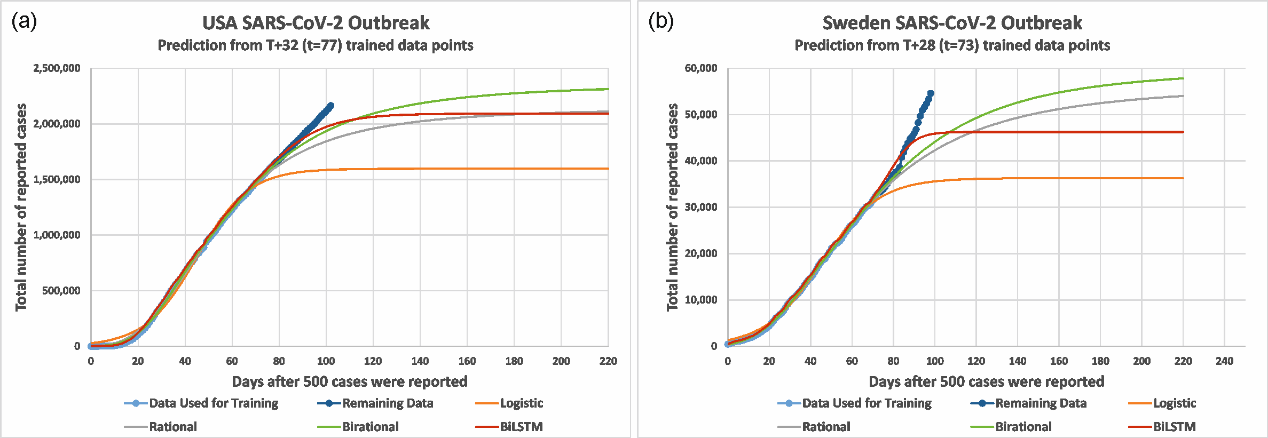


**Figure 2**. SARS-CoV-2 virus infection for **(a)** USA and **(b)** Sweden: Predicted vs Actual for the total cumulative number of individuals reported to be infected as a function of days after the day that 500 cases were reported. The prediction fits were obtained using training data up to May 24, 2020. The dark blue curves correspond to the actual test data reported after May 25 up to June 17, 2020. These figures illustrate clearly the failure of both the analytical formulas and the deep learning network to make accurate predictions.
